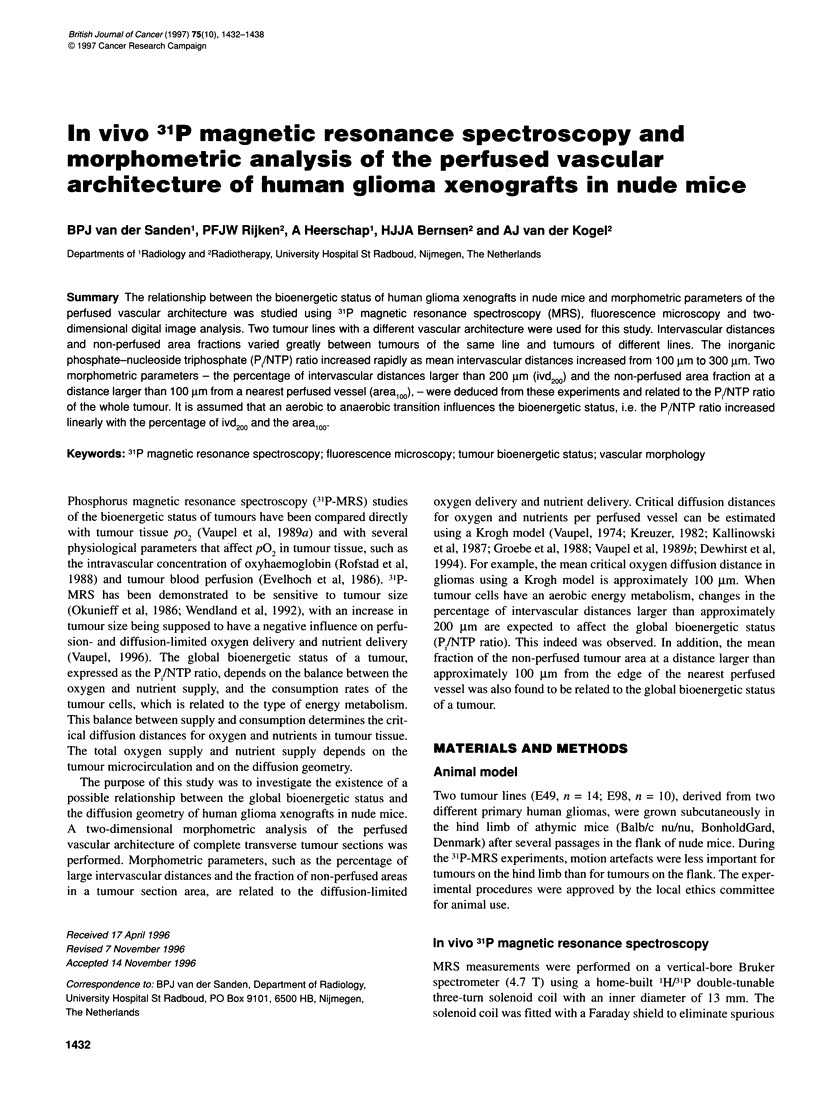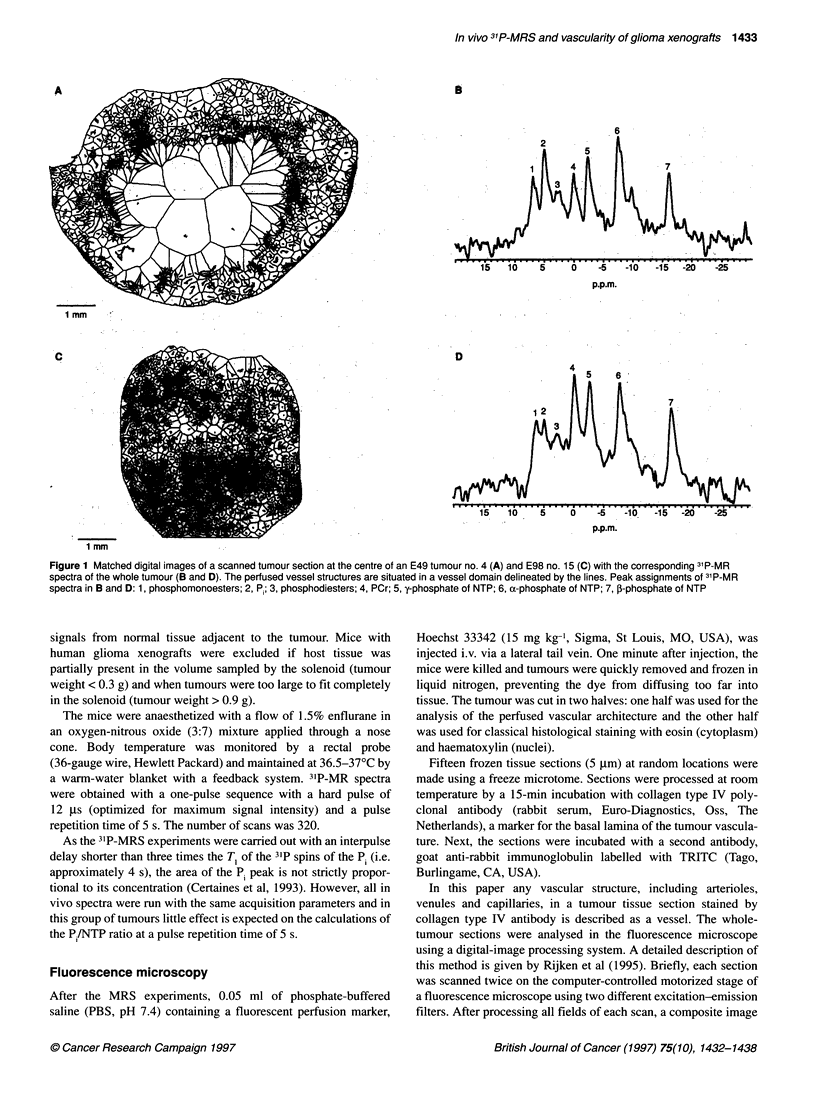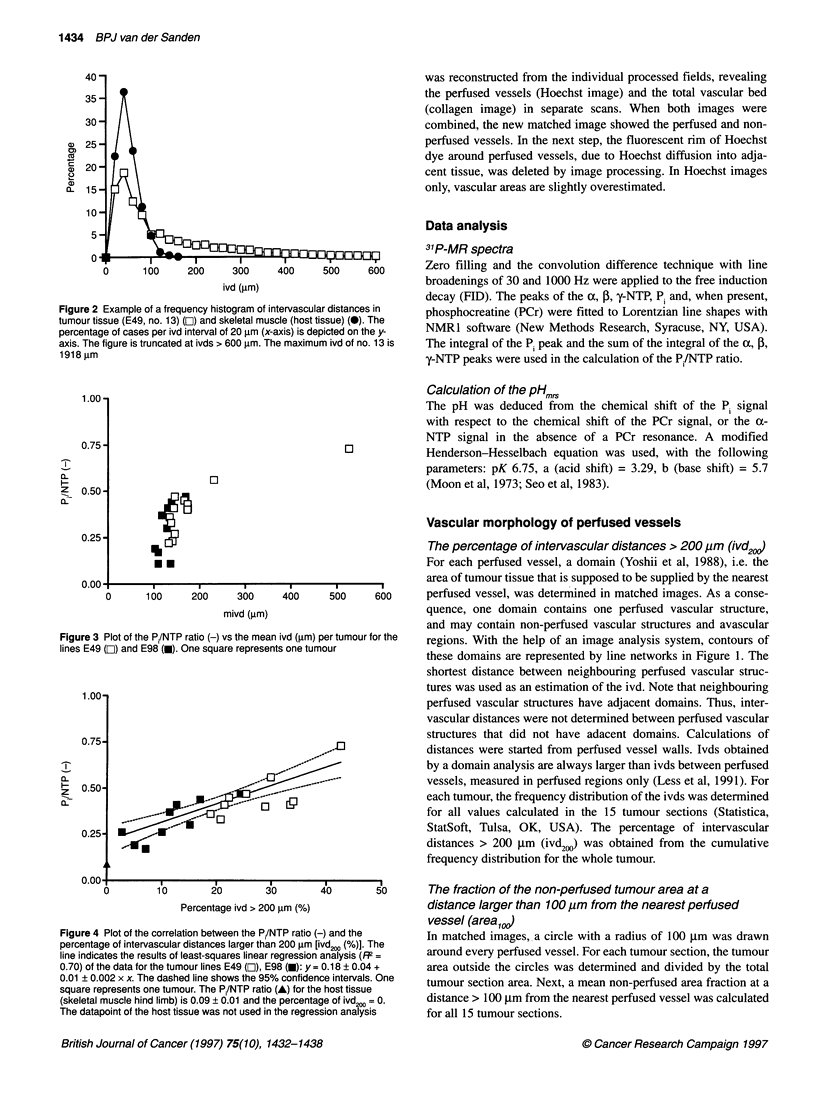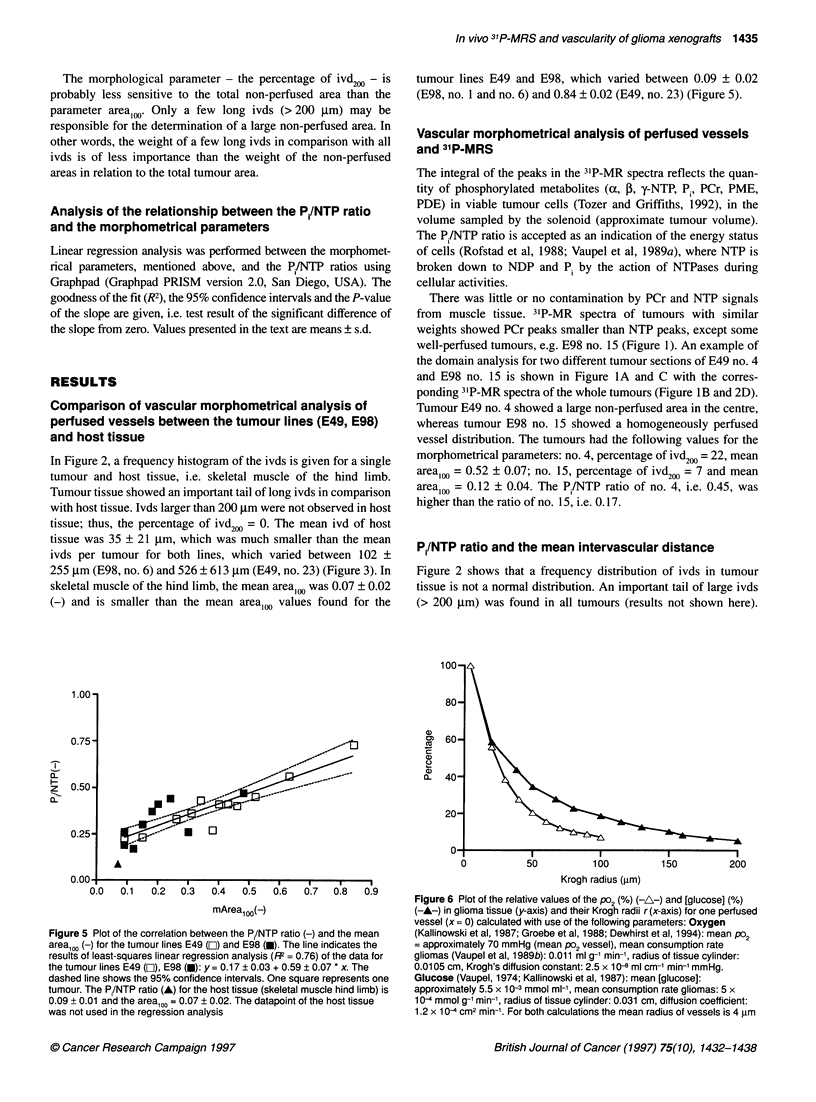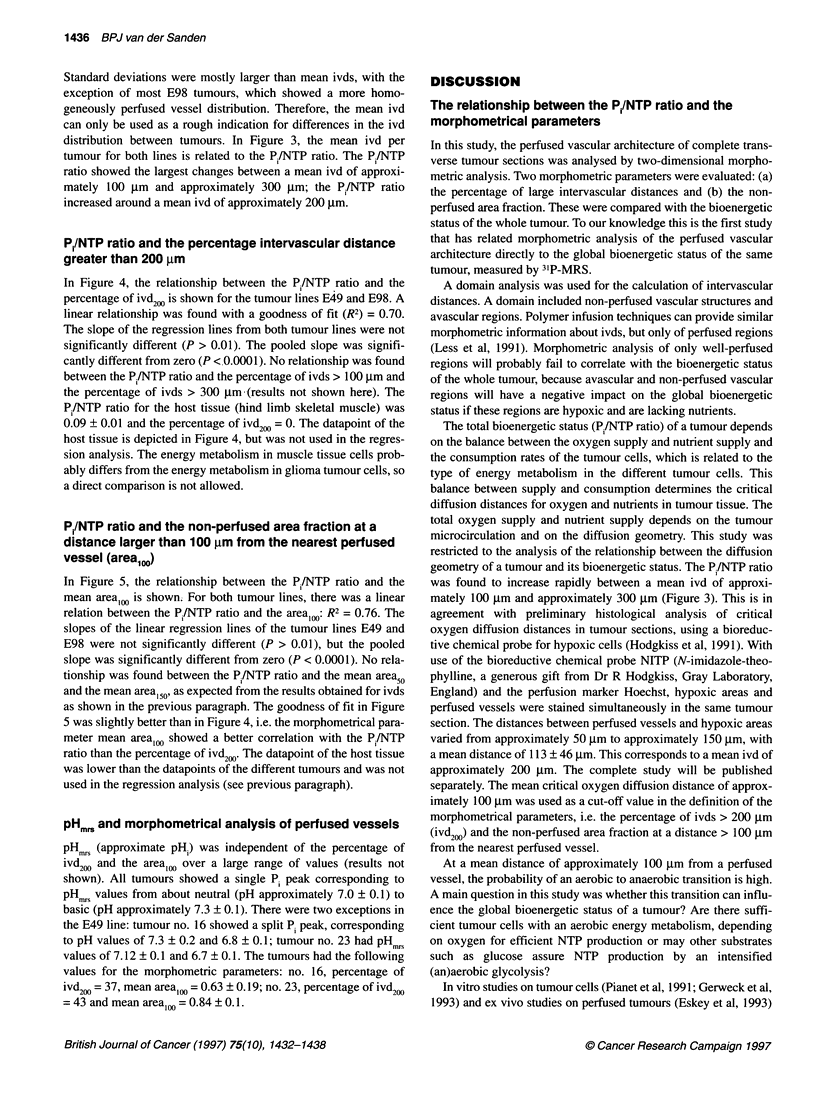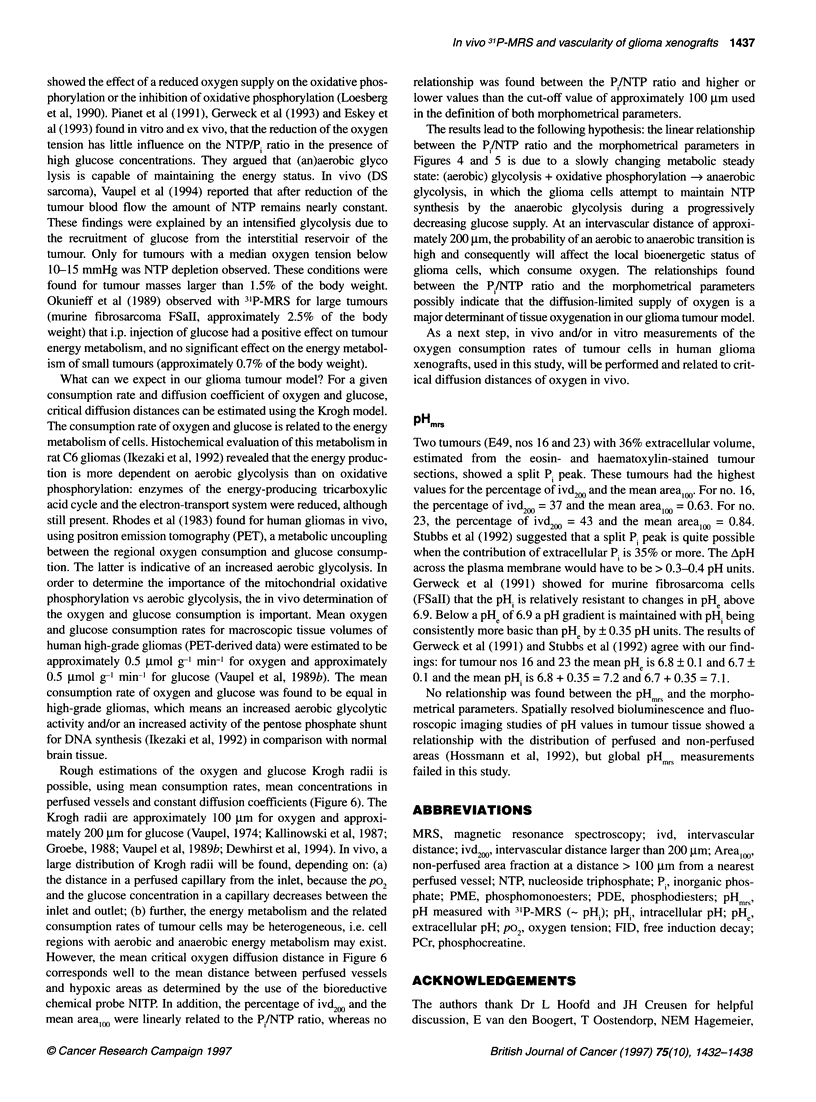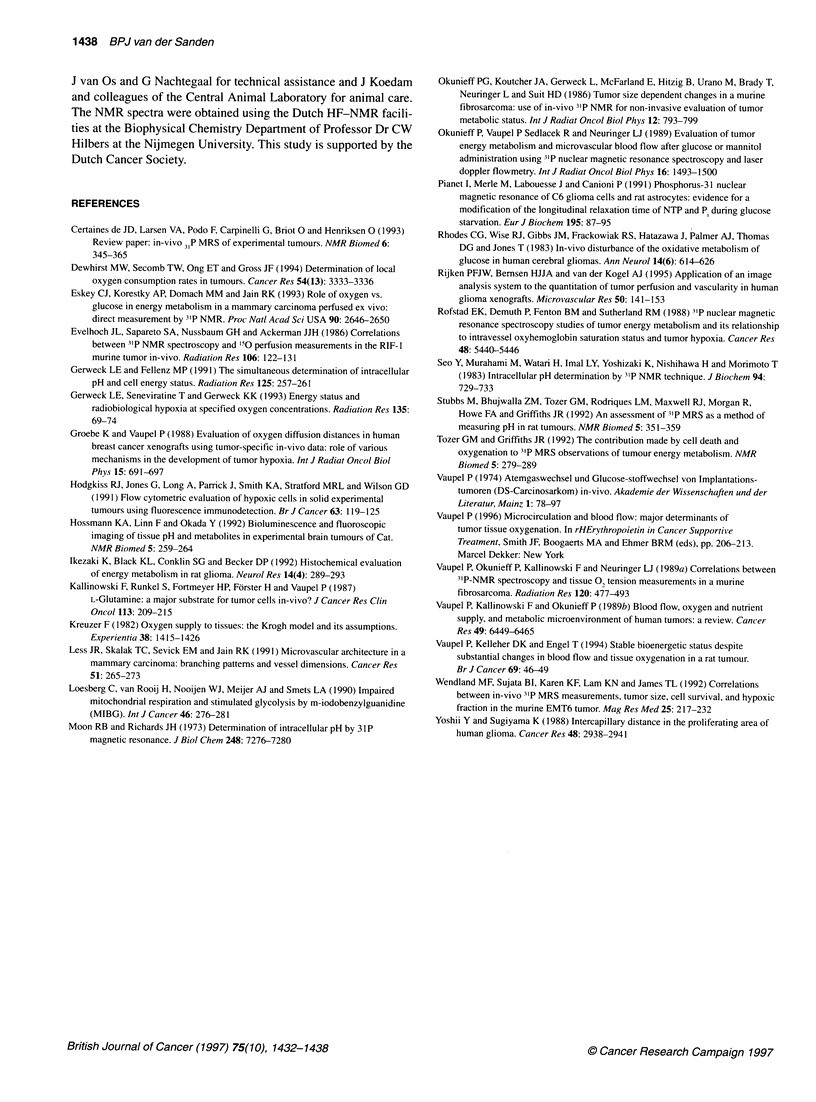# In vivo (31)P magnetic resonance spectroscopy and morphometric analysis of the perfused vascular architecture of human glioma xenografts in nude mice.

**DOI:** 10.1038/bjc.1997.246

**Published:** 1997

**Authors:** B. P. van der Sanden, P. F. Rijken, A. Heerschap, H. J. Bernsen, A. J. van der Kogel

**Affiliations:** Department of Radiology, University Hospital St Radboud, Nijmegen, The Netherlands.

## Abstract

The relationship between the bioenergetic status of human glioma xenografts in nude mice and morphometric parameters of the perfused vascular architecture was studied using (31)P magnetic resonance spectroscopy (MRS), fluorescence microscopy and two-dimensional digital image analysis. Two tumour lines with a different vascular architecture were used for this study. Intervascular distances and non-perfused area fractions varied greatly between tumours of the same line and tumours of different lines. The inorganic phosphate-nucleoside triphosphate (P(i)/NTP) ratio increased rapidly as mean intervascular distances increased from 100 microm to 300 microm. Two morphometric parameters - the percentage of intervascular distances larger than 200 microm (ivd200) and the non-perfused area fraction at a distance larger than 100 microm from a nearest perfused vessel (area100), - were deduced from these experiments and related to the P(i)/NTP ratio of the whole tumour. It is assumed that an aerobic to anaerobic transition influences the bioenergetic status, i.e. the P(i)/NTP ratio increased linearly with the percentage of ivd200 and the area100.


British Journal of Cancer (1997) 75(10), 1432–1438
© 1997 Cancer Research Campaign

### In vivo $^{31}P$ magnetic resonance spectroscopy and morphometric analysis of the perfused vascular architecture of human glioma xenografts in nude mice

**BPJ van der Sanden[1], PFJW Rijken[2], A Heerschap[1], HJJA Bernsen[2] and AJ van der Kogel[2]**

Departments of [1]Radiology and [2]Radiotherapy, University Hospital St Radboud, Nijmegen, The Netherlands

**Summary** The relationship between the bioenergetic status of human glioma xenografts in nude mice and morphometric parameters of the perfused vascular architecture was studied using $^{31}P$ magnetic resonance spectroscopy (MRS), fluorescence microscopy and two-dimensional digital image analysis. Two tumour lines with a different vascular architecture were used for this study. Intervascular distances and non-perfused area fractions varied greatly between tumours of the same line and tumours of different lines. The inorganic phosphate–nucleoside triphosphate ($P_i$/NTP) ratio increased rapidly as mean intervascular distances increased from 100 μm to 300 μm. Two morphometric parameters – the percentage of intervascular distances larger than 200 μm ($ivd_{200}$) and the non-perfused area fraction at a distance larger than 100 μm from a nearest perfused vessel ($area_{100}$), – were deduced from these experiments and related to the $P_i$/NTP ratio of the whole tumour. It is assumed that an aerobic to anaerobic transition influences the bioenergetic status, i.e. the $P_i$/NTP ratio increased linearly with the percentage of $ivd_{200}$ and the $area_{100}$.

**Keywords:** $^{31}P$ magnetic resonance spectroscopy; fluorescence microscopy; tumour bioenergetic status; vascular morphology

Phosphorus magnetic resonance spectroscopy ($^{31}P$-MRS) studies of the bioenergetic status of tumours have been compared directly with tumour tissue $pO_2$ (Vaupel et al, 1989*a*) and with several physiological parameters that affect $pO_2$ in tumour tissue, such as the intravascular concentration of oxyhaemoglobin (Rofstad et al, 1988) and tumour blood perfusion (Evelhoch et al, 1986). $^{31}P$-MRS has been demonstrated to be sensitive to tumour size (Okunieff et al, 1986; Wendland et al, 1992), with an increase in tumour size being supposed to have a negative influence on perfusion- and diffusion-limited oxygen delivery and nutrient delivery (Vaupel, 1996). The global bioenergetic status of a tumour, expressed as the $P_i$/NTP ratio, depends on the balance between the oxygen and nutrient supply, and the consumption rates of the tumour cells, which is related to the type of energy metabolism. This balance between supply and consumption determines the critical diffusion distances for oxygen and nutrients in tumour tissue. The total oxygen supply and nutrient supply depends on the tumour microcirculation and on the diffusion geometry.

The purpose of this study was to investigate the existence of a possible relationship between the global bioenergetic status and the diffusion geometry of human glioma xenografts in nude mice. A two-dimensional morphometric analysis of the perfused vascular architecture of complete transverse tumour sections was performed. Morphometric parameters, such as the percentage of large intervascular distances and the fraction of non-perfused areas in a tumour section area, are related to the diffusion-limited oxygen delivery and nutrient delivery. Critical diffusion distances for oxygen and nutrients per perfused vessel can be estimated using a Krogh model (Vaupel, 1974; Kreuzer, 1982; Kallinowski et al, 1987; Groebe et al, 1988; Vaupel et al, 1989*b*; Dewhirst et al, 1994). For example, the mean critical oxygen diffusion distance in gliomas using a Krogh model is approximately 100 μm. When tumour cells have an aerobic energy metabolism, changes in the percentage of intervascular distances larger than approximately 200 μm are expected to affect the global bioenergetic status ($P_i$/NTP ratio). This indeed was observed. In addition, the mean fraction of the non-perfused tumour area at a distance larger than approximately 100 μm from the edge of the nearest perfused vessel was also found to be related to the global bioenergetic status of a tumour.

*Received 17 April 1996*
*Revised 7 November 1996*
*Accepted 14 November 1996*

*Correspondence to:* BPJ van der Sanden, Department of Radiology, University Hospital St Radboud, PO Box 9101, 6500 HB, Nijmegen, The Netherlands

### MATERIALS AND METHODS

### Animal model

Two tumour lines (E49, *n* = 14; E98, *n* = 10), derived from two different primary human gliomas, were grown subcutaneously in the hind limb of athymic mice (Balb/c nu/nu, BonholdGard, Denmark) after several passages in the flank of nude mice. During the $^{31}P$-MRS experiments, motion artefacts were less important for tumours on the hind limb than for tumours on the flank. The experimental procedures were approved by the local ethics committee for animal use.

### In vivo $^{31}P$ magnetic resonance spectroscopy

MRS measurements were performed on a vertical-bore Bruker spectrometer (4.7 T) using a home-built $^{1}H/^{31}P$ double-tunable three-turn solenoid coil with an inner diameter of 13 mm. The solenoid coil was fitted with a Faraday shield to eliminate spurious

**Figure 1** Matched digital images of a scanned tumour section at the centre of an E49 tumour no. 4 (**A**) and E98 no. 15 (**C**) with the corresponding $^{31}$P-MR spectra of the whole tumour (**B** and **D**). The perfused vessel structures are situated in a vessel domain delineated by the lines. Peak assignments of $^{31}$P-MR spectra in **B** and **D**: 1, phosphomonoesters; 2, $P_i$; 3, phosphodiesters; 4, PCr; 5, γ-phosphate of NTP; 6, α-phosphate of NTP; 7, β-phosphate of NTP

signals from normal tissue adjacent to the tumour. Mice with human glioma xenografts were excluded if host tissue was partially present in the volume sampled by the solenoid (tumour weight < 0.3 g) and when tumours were too large to fit completely in the solenoid (tumour weight > 0.9 g).

The mice were anaesthetized with a flow of 1.5% enflurane in an oxygen-nitrous oxide (3:7) mixture applied through a nose cone. Body temperature was monitored by a rectal probe (36-gauge wire, Hewlett Packard) and maintained at 36.5–37°C by a warm-water blanket with a feedback system. $^{31}$P-MR spectra were obtained with a one-pulse sequence with a hard pulse of 12 μs (optimized for maximum signal intensity) and a pulse repetition time of 5 s. The number of scans was 320.

As the $^{31}$P-MRS experiments were carried out with an interpulse delay shorter than three times the $T_1$ of the $^{31}$P spins of the $P_i$ (i.e. approximately 4 s), the area of the $P_i$ peak is not strictly proportional to its concentration (Certaines et al, 1993). However, all in vivo spectra were run with the same acquisition parameters and in this group of tumours little effect is expected on the calculations of the $P_i$/NTP ratio at a pulse repetition time of 5 s.

### Fluorescence microscopy

After the MRS experiments, 0.05 ml of phosphate-buffered saline (PBS, pH 7.4) containing a fluorescent perfusion marker, Hoechst 33342 (15 mg kg$^{-1}$, Sigma, St Louis, MO, USA), was injected i.v. via a lateral tail vein. One minute after injection, the mice were killed and tumours were quickly removed and frozen in liquid nitrogen, preventing the dye from diffusing too far into tissue. The tumour was cut in two halves: one half was used for the analysis of the perfused vascular architecture and the other half was used for classical histological staining with eosin (cytoplasm) and haematoxylin (nuclei).

Fifteen frozen tissue sections (5 μm) at random locations were made using a freeze microtome. Sections were processed at room temperature by a 15-min incubation with collagen type IV polyclonal antibody (rabbit serum, Euro-Diagnostics, Oss, The Netherlands), a marker for the basal lamina of the tumour vasculature. Next, the sections were incubated with a second antibody, goat anti-rabbit immunoglobulin labelled with TRITC (Tago, Burlingame, CA, USA).

In this paper any vascular structure, including arterioles, venules and capillaries, in a tumour tissue section stained by collagen type IV antibody is described as a vessel. The whole-tumour sections were analysed in the fluorescence microscope using a digital-image processing system. A detailed description of this method is given by Rijken et al (1995). Briefly, each section was scanned twice on the computer-controlled motorized stage of a fluorescence microscope using two different excitation–emission filters. After processing all fields of each scan, a composite image

© Cancer Research Campaign 1997 

**Figure 2** Example of a frequency histogram of intervascular distances in tumour tissue (E49, no. 13) (□) and skeletal muscle (host tissue) (●). The percentage of cases per ivd interval of 20 μm (*x*-axis) is depicted on the *y*-axis. The figure is truncated at ivds > 600 μm. The maximum ivd of no. 13 is 1918 μm

**Figure 3** Plot of the $P_i$/NTP ratio (–) vs the mean ivd (μm) per tumour for the lines E49 (□) and E98 (■). One square represents one tumour

**Figure 4** Plot of the correlation between the $P_i$/NTP ratio (–) and the percentage of intervascular distances larger than 200 μm [$ivd_{200}$ (%)]. The line indicates the results of least-squares linear regression analysis ($R^2$ = 0.70) of the data for the tumour lines E49 (□), E98 (■): $y = 0.18 \pm 0.04 + 0.01 \pm 0.002 \times x$. The dashed line shows the 95% confidence intervals. One square represents one tumour. The $P_i$/NTP ratio (▲) for the host tissue (skeletal muscle hind limb) is 0.09 ± 0.01 and the percentage of $ivd_{200}$ = 0. The datapoint of the host tissue was not used in the regression analysis was reconstructed from the individual processed fields, revealing the perfused vessels (Hoechst image) and the total vascular bed (collagen image) in separate scans. When both images were combined, the new matched image showed the perfused and non-perfused vessels. In the next step, the fluorescent rim of Hoechst dye around perfused vessels, due to Hoechst diffusion into adjacent tissue, was deleted by image processing. In Hoechst images only, vascular areas are slightly overestimated.

### Data analysis

### *³¹P-MR spectra*

Zero filling and the convolution difference technique with line broadenings of 30 and 1000 Hz were applied to the free induction decay (FID). The peaks of the α, β, γ-NTP, $P_i$ and, when present, phosphocreatine (PCr) were fitted to Lorentzian line shapes with NMR1 software (New Methods Research, Syracuse, NY, USA). The integral of the $P_i$ peak and the sum of the integral of the α, β, γ-NTP peaks were used in the calculation of the $P_i$/NTP ratio.

### *Calculation of the $pH_{mrs}$*

The pH was deduced from the chemical shift of the $P_i$ signal with respect to the chemical shift of the PCr signal, or the α-NTP signal in the absence of a PCr resonance. A modified Henderson–Hesselbach equation was used, with the following parameters: p*K* 6.75, a (acid shift) = 3.29, b (base shift) = 5.7 (Moon et al, 1973; Seo et al, 1983).

### Vascular morphology of perfused vessels

### *The percentage of intervascular distances > 200 μm ($ivd_{200}$)*

For each perfused vessel, a domain (Yoshii et al, 1988), i.e. the area of tumour tissue that is supposed to be supplied by the nearest perfused vessel, was determined in matched images. As a consequence, one domain contains one perfused vascular structure, and may contain non-perfused vascular structures and avascular regions. With the help of an image analysis system, contours of these domains are represented by line networks in Figure 1. The shortest distance between neighbouring perfused vascular structures was used as an estimation of the ivd. Note that neighbouring perfused vascular structures have adjacent domains. Thus, intervascular distances were not determined between perfused vascular structures that did not have adacent domains. Calculations of distances were started from perfused vessel walls. Ivds obtained by a domain analysis are always larger than ivds between perfused vessels, measured in perfused regions only (Less et al, 1991). For each tumour, the frequency distribution of the ivds was determined for all values calculated in the 15 tumour sections (Statistica, StatSoft, Tulsa, OK, USA). The percentage of intervascular distances > 200 μm ($ivd_{200}$) was obtained from the cumulative frequency distribution for the whole tumour.

### *The fraction of the non-perfused tumour area at a distance larger than 100 μm from the nearest perfused vessel ($area_{100}$)*

In matched images, a circle with a radius of 100 μm was drawn around every perfused vessel. For each tumour section, the tumour area outside the circles was determined and divided by the total tumour section area. Next, a mean non-perfused area fraction at a distance > 100 μm from the nearest perfused vessel was calculated for all 15 tumour sections.

 © Cancer Research Campaign 1997

The morphological parameter – the percentage of $ivd_{200}$ – is probably less sensitive to the total non-perfused area than the parameter $area_{100}$. Only a few long ivds (> 200 μm) may be responsible for the determination of a large non-perfused area. In other words, the weight of a few long ivds in comparison with all ivds is of less importance than the weight of the non-perfused areas in relation to the total tumour area.

### Analysis of the relationship between the $P_i$/NTP ratio and the morphometrical parameters

Linear regression analysis was performed between the morphometrical parameters, mentioned above, and the $P_i$/NTP ratios using Graphpad (Graphpad PRISM version 2.0, San Diego, USA). The goodness of the fit ($R^2$), the 95% confidence intervals and the $P$-value of the slope are given, i.e. test result of the significant difference of the slope from zero. Values presented in the text are means ± s.d.

### RESULTS

### Comparison of vascular morphometrical analysis of perfused vessels between the tumour lines (E49, E98) and host tissue

In Figure 2, a frequency histogram of the ivds is given for a single tumour and host tissue, i.e. skeletal muscle of the hind limb. Tumour tissue showed an important tail of long ivds in comparison with host tissue. Ivds larger than 200 μm were not observed in host tissue; thus, the percentage of $ivd_{200}$ = 0. The mean ivd of host tissue was 35 ± 21 μm, which was much smaller than the mean ivds per tumour for both lines, which varied between 102 ± 255 μm (E98, no. 6) and 526 ± 613 μm (E49, no. 23) (Figure 3). In skeletal muscle of the hind limb, the mean $area_{100}$ was 0.07 ± 0.02 (–) and is smaller than the mean $area_{100}$ values found for the tumour lines E49 and E98, which varied between 0.09 ± 0.02 (E98, no. 1 and no. 6) and 0.84 ± 0.02 (E49, no. 23) (Figure 5).

### Vascular morphometrical analysis of perfused vessels and $^{31}$P-MRS

The integral of the peaks in the $^{31}$P-MR spectra reflects the quantity of phosphorylated metabolites (α, β, γ-NTP, $P_i$, PCr, PME, PDE) in viable tumour cells (Tozer and Griffiths, 1992), in the volume sampled by the solenoid (approximate tumour volume). The $P_i$/NTP ratio is accepted as an indication of the energy status of cells (Rofstad et al, 1988; Vaupel et al, 1989*a*), where NTP is broken down to NDP and $P_i$ by the action of NTPases during cellular activities.

There was little or no contamination by PCr and NTP signals from muscle tissue. $^{31}$P-MR spectra of tumours with similar weights showed PCr peaks smaller than NTP peaks, except some well-perfused tumours, e.g. E98 no. 15 (Figure 1). An example of the domain analysis for two different tumour sections of E49 no. 4 and E98 no. 15 is shown in Figure 1A and C with the corresponding $^{31}$P-MR spectra of the whole tumours (Figure 1B and 2D). Tumour E49 no. 4 showed a large non-perfused area in the centre, whereas tumour E98 no. 15 showed a homogeneously perfused vessel distribution. The tumours had the following values for the morphometrical parameters: no. 4, percentage of $ivd_{200}$ = 22, mean $area_{100}$ = 0.52 ± 0.07; no. 15, percentage of $ivd_{200}$ = 7 and mean $area_{100}$ = 0.12 ± 0.04. The $P_i$/NTP ratio of no. 4, i.e. 0.45, was higher than the ratio of no. 15, i.e. 0.17.

### $P_i$/NTP ratio and the mean intervascular distance

Figure 2 shows that a frequency distribution of ivds in tumour tissue is not a normal distribution. An important tail of large ivds (> 200 μm) was found in all tumours (results not shown here).

**Figure 5** Plot of the correlation between the $P_i$/NTP ratio (–) and the mean $area_{100}$ (–) for the tumour lines E49 (□) and E98 (■). The line indicates the results of least-squares linear regression analysis ($R^2$ = 0.76) of the data for the tumour lines E49 (□), E98 (■): $y = 0.17 \pm 0.03 + 0.59 \pm 0.07 * x$. The dashed line shows the 95% confidence intervals. One square represents one tumour. The $P_i$/NTP ratio (▲) for the host tissue (skeletal muscle hind limb) is 0.09 ± 0.01 and the $area_{100}$ = 0.07 ± 0.02. The datapoint of the host tissue was not used in the regression analysis

**Figure 6** Plot of the relative values of the $pO_2$ (%) (–△–) and [glucose] (%) (–▲–) in glioma tissue ($y$-axis) and their Krogh radii $r$ ($x$-axis) for one perfused vessel ($x$ = 0) calculated with use of the following parameters: **Oxygen** (Kallinowski et al, 1987; Groebe et al, 1988; Dewhirst et al, 1994): mean $pO_2$ = approximately 70 mmHg (mean $pO_2$ vessel), mean consumption rate gliomas (Vaupel et al, 1989*b*): 0.011 ml g$^{-1}$ min$^{-1}$, radius of tissue cylinder: 0.0105 cm, Krogh's diffusion constant: 2.5 × 10$^{-8}$ ml cm$^{-1}$ min$^{-1}$ mmHg. **Glucose** (Vaupel, 1974; Kallinowski et al, 1987): mean [glucose]: approximately 5.5 × 10$^{-3}$ mmol ml$^{-1}$, mean consumption rate gliomas: 5 × 10$^{-4}$ mmol g$^{-1}$ min$^{-1}$, radius of tissue cylinder: 0.031 cm, diffusion coefficient: 1.2 × 10$^{-4}$ cm$^2$ min$^{-1}$. For both calculations the mean radius of vessels is 4 μm

© Cancer Research Campaign 1997

Standard deviations were mostly larger than mean ivds, with the exception of most E98 tumours, which showed a more homogeneously perfused vessel distribution. Therefore, the mean ivd can only be used as a rough indication for differences in the ivd distribution between tumours. In Figure 3, the mean ivd per tumour for both lines is related to the $P_i$/NTP ratio. The $P_i$/NTP ratio showed the largest changes between a mean ivd of approximately 100 μm and approximately 300 μm; the $P_i$/NTP ratio increased around a mean ivd of approximately 200 μm.

### $P_i$/NTP ratio and the percentage intervascular distance greater than 200 μm

In Figure 4, the relationship between the $P_i$/NTP ratio and the percentage of $ivd_{200}$ is shown for the tumour lines E49 and E98. A linear relationship was found with a goodness of fit ($R^2$) = 0.70. The slope of the regression lines from both tumour lines were not significantly different ($P > 0.01$). The pooled slope was significantly different from zero ($P < 0.0001$). No relationship was found between the $P_i$/NTP ratio and the percentage of ivds > 100 μm and the percentage of ivds > 300 μm (results not shown here). The $P_i$/NTP ratio for the host tissue (hind limb skeletal muscle) was 0.09 ± 0.01 and the percentage of $ivd_{200}$ = 0. The datapoint of the host tissue is depicted in Figure 4, but was not used in the regression analysis. The energy metabolism in muscle tissue cells probably differs from the energy metabolism in glioma tumour cells, so a direct comparison is not allowed.

### $P_i$/NTP ratio and the non-perfused area fraction at a distance larger than 100 μm from the nearest perfused vessel ($area_{100}$)

In Figure 5, the relationship between the $P_i$/NTP ratio and the mean $area_{100}$ is shown. For both tumour lines, there was a linear relation between the $P_i$/NTP ratio and the $area_{100}$: $R^2$ = 0.76. The slopes of the linear regression lines of the tumour lines E49 and E98 were not significantly different ($P > 0.01$), but the pooled slope was significantly different from zero ($P < 0.0001$). No relationship was found between the $P_i$/NTP ratio and the mean $area_{50}$ and the mean $area_{150}$, as expected from the results obtained for ivds as shown in the previous paragraph. The goodness of fit in Figure 5 was slightly better than in Figure 4, i.e. the morphometrical parameter mean $area_{100}$ showed a better correlation with the $P_i$/NTP ratio than the percentage of $ivd_{200}$. The datapoint of the host tissue was lower than the datapoints of the different tumours and was not used in the regression analysis (see previous paragraph).

### $pH_{mrs}$ and morphometrical analysis of perfused vessels

$pH_{mrs}$ (approximate $pH_i$) was independent of the percentage of $ivd_{200}$ and the $area_{100}$ over a large range of values (results not shown). All tumours showed a single $P_i$ peak corresponding to $pH_{mrs}$ values from about neutral (pH approximately 7.0 ± 0.1) to basic (pH approximately 7.3 ± 0.1). There were two exceptions in the E49 line: tumour no. 16 showed a split $P_i$ peak, corresponding to pH values of 7.3 ± 0.2 and 6.8 ± 0.1; tumour no. 23 had $pH_{mrs}$ values of 7.12 ± 0.1 and 6.7 ± 0.1. The tumours had the following values for the morphometric parameters: no. 16, percentage of $ivd_{200}$ = 37, mean $area_{100}$ = 0.63 ± 0.19; no. 23, percentage of $ivd_{200}$ = 43 and mean $area_{100}$ = 0.84 ± 0.1.

### DISCUSSION

### The relationship between the $P_i$/NTP ratio and the morphometrical parameters

In this study, the perfused vascular architecture of complete transverse tumour sections was analysed by two-dimensional morphometric analysis. Two morphometric parameters were evaluated: (a) the percentage of large intervascular distances and (b) the non-perfused area fraction. These were compared with the bioenergetic status of the whole tumour. To our knowledge this is the first study that has related morphometric analysis of the perfused vascular architecture directly to the global bioenergetic status of the same tumour, measured by $^{31}$P-MRS.

A domain analysis was used for the calculation of intervascular distances. A domain included non-perfused vascular structures and avascular regions. Polymer infusion techniques can provide similar morphometric information about ivds, but only of perfused regions (Less et al, 1991). Morphometric analysis of only well-perfused regions will probably fail to correlate with the bioenergetic status of the whole tumour, because avascular and non-perfused vascular regions will have a negative impact on the global bioenergetic status if these regions are hypoxic and are lacking nutrients.

The total bioenergetic status ($P_i$/NTP ratio) of a tumour depends on the balance between the oxygen supply and nutrient supply and the consumption rates of the tumour cells, which is related to the type of energy metabolism in the different tumour cells. This balance between supply and consumption determines the critical diffusion distances for oxygen and nutrients in tumour tissue. The total oxygen supply and nutrient supply depends on the tumour microcirculation and on the diffusion geometry. This study was restricted to the analysis of the relationship between the diffusion geometry of a tumour and its bioenergetic status. The $P_i$/NTP ratio was found to increase rapidly between a mean ivd of approximately 100 μm and approximately 300 μm (Figure 3). This is in agreement with preliminary histological analysis of critical oxygen diffusion distances in tumour sections, using a bioreductive chemical probe for hypoxic cells (Hodgkiss et al, 1991). With use of the bioreductive chemical probe NITP (*N*-imidazole-theophylline, a generous gift from Dr R Hodgkiss, Gray Laboratory, England) and the perfusion marker Hoechst, hypoxic areas and perfused vessels were stained simultaneously in the same tumour section. The distances between perfused vessels and hypoxic areas varied from approximately 50 μm to approximately 150 μm, with a mean distance of 113 ± 46 μm. This corresponds to a mean ivd of approximately 200 μm. The complete study will be published separately. The mean critical oxygen diffusion distance of approximately 100 μm was used as a cut-off value in the definition of the morphometrical parameters, i.e. the percentage of ivds > 200 μm ($ivd_{200}$) and the non-perfused area fraction at a distance > 100 μm from the nearest perfused vessel.

At a mean distance of approximately 100 μm from a perfused vessel, the probability of an aerobic to anaerobic transition is high. A main question in this study was whether this transition can influence the global bioenergetic status of a tumour? Are there sufficient tumour cells with an aerobic energy metabolism, depending on oxygen for efficient NTP production or may other substrates such as glucose assure NTP production by an intensified (an)aerobic glycolysis?

In vitro studies on tumour cells (Pianet et al, 1991; Gerweck et al, 1993) and ex vivo studies on perfused tumours (Eskey et al, 1993)

© Cancer Research Campaign 1997

showed the effect of a reduced oxygen supply on the oxidative phosphorylation or the inhibition of oxidative phosphorylation (Loesberg et al, 1990). Pianet et al (1991), Gerweck et al (1993) and Eskey et al (1993) found in vitro and ex vivo, that the reduction of the oxygen tension has little influence on the NTP/$P_i$ ratio in the presence of high glucose concentrations. They argued that (an)aerobic glyco lysis is capable of maintaining the energy status. In vivo (DS sarcoma), Vaupel et al (1994) reported that after reduction of the tumour blood flow the amount of NTP remains nearly constant. These findings were explained by an intensified glycolysis due to the recruitment of glucose from the interstitial reservoir of the tumour. Only for tumours with a median oxygen tension below 10–15 mmHg was NTP depletion observed. These conditions were found for tumour masses larger than 1.5% of the body weight. Okunieff et al (1989) observed with $^{31}$P-MRS for large tumours (murine fibrosarcoma FSaII, approximately 2.5% of the body weight) that i.p. injection of glucose had a positive effect on tumour energy metabolism, and no significant effect on the energy metabolism of small tumours (approximately 0.7% of the body weight).

What can we expect in our glioma tumour model? For a given consumption rate and diffusion coefficient of oxygen and glucose, critical diffusion distances can be estimated using the Krogh model. The consumption rate of oxygen and glucose is related to the energy metabolism of cells. Histochemical evaluation of this metabolism in rat C6 gliomas (Ikezaki et al, 1992) revealed that the energy production is more dependent on aerobic glycolysis than on oxidative phosphorylation: enzymes of the energy-producing tricarboxylic acid cycle and the electron-transport system were reduced, although still present. Rhodes et al (1983) found for human gliomas in vivo, using positron emission tomography (PET), a metabolic uncoupling between the regional oxygen consumption and glucose consumption. The latter is indicative of an increased aerobic glycolysis. In order to determine the importance of the mitochondrial oxidative phosphorylation vs aerobic glycolysis, the in vivo determination of the oxygen and glucose consumption is important. Mean oxygen and glucose consumption rates for macroscopic tissue volumes of human high-grade gliomas (PET-derived data) were estimated to be approximately 0.5 μmol $g^{-1}$ $min^{-1}$ for oxygen and approximately 0.5 μmol $g^{-1}$ $min^{-1}$ for glucose (Vaupel et al, 1989*b*). The mean consumption rate of oxygen and glucose was found to be equal in high-grade gliomas, which means an increased aerobic glycolytic activity and/or an increased activity of the pentose phosphate shunt for DNA synthesis (Ikezaki et al, 1992) in comparison with normal brain tissue.

Rough estimations of the oxygen and glucose Krogh radii is possible, using mean consumption rates, mean concentrations in perfused vessels and constant diffusion coefficients (Figure 6). The Krogh radii are approximately 100 μm for oxygen and approximately 200 μm for glucose (Vaupel, 1974; Kallinowski et al, 1987; Groebe, 1988; Vaupel et al, 1989*b*; Dewhirst et al, 1994). In vivo, a large distribution of Krogh radii will be found, depending on: (a) the distance in a perfused capillary from the inlet, because the $po_2$ and the glucose concentration in a capillary decreases between the inlet and outlet; (b) further, the energy metabolism and the related consumption rates of tumour cells may be heterogeneous, i.e. cell regions with aerobic and anaerobic energy metabolism may exist. However, the mean critical oxygen diffusion distance in Figure 6 corresponds well to the mean distance between perfused vessels and hypoxic areas as determined by the use of the bioreductive chemical probe NITP. In addition, the percentage of $ivd_{200}$ and the mean $area_{100}$ were linearly related to the $P_i$/NTP ratio, whereas no relationship was found between the $P_i$/NTP ratio and higher or lower values than the cut-off value of approximately 100 μm used in the definition of both morphometrical parameters.

The results lead to the following hypothesis: the linear relationship between the $P_i$/NTP ratio and the morphometrical parameters in Figures 4 and 5 is due to a slowly changing metabolic steady state: (aerobic) glycolysis + oxidative phosphorylation → anaerobic glycolysis, in which the glioma cells attempt to maintain NTP synthesis by the anaerobic glycolysis during a progressively decreasing glucose supply. At an intervascular distance of approximately 200 μm, the probability of an aerobic to anaerobic transition is high and consequently will affect the local bioenergetic status of glioma cells, which consume oxygen. The relationships found between the $P_i$/NTP ratio and the morphometrical parameters possibly indicate that the diffusion-limited supply of oxygen is a major determinant of tissue oxygenation in our glioma tumour model.

As a next step, in vivo and/or in vitro measurements of the oxygen consumption rates of tumour cells in human glioma xenografts, used in this study, will be performed and related to critical diffusion distances of oxygen in vivo.

### $pH_{mrs}$

Two tumours (E49, nos 16 and 23) with 36% extracellular volume, estimated from the eosin- and haematoxylin-stained tumour sections, showed a split $P_i$ peak. These tumours had the highest values for the percentage of $ivd_{200}$ and the mean $area_{100}$. For no. 16, the percentage of $ivd_{200}$ = 37 and the mean $area_{100}$ = 0.63. For no. 23, the percentage of $ivd_{200}$ = 43 and the mean $area_{100}$ = 0.84. Stubbs et al (1992) suggested that a split $P_i$ peak is quite possible when the contribution of extracellular $P_i$ is 35% or more. The ΔpH across the plasma membrane would have to be > 0.3–0.4 pH units. Gerweck et al (1991) showed for murine fibrosarcoma cells (FSaII) that the $pH_i$ is relatively resistant to changes in $pH_e$ above 6.9. Below a $pH_e$ of 6.9 a pH gradient is maintained with $pH_i$ being consistently more basic than $pH_e$ by ± 0.35 pH units. The results of Gerweck et al (1991) and Stubbs et al (1992) agree with our findings: for tumour nos 16 and 23 the mean $pH_e$ is 6.8 ± 0.1 and 6.7 ± 0.1 and the mean $pH_i$ is 6.8 + 0.35 = 7.2 and 6.7 + 0.35 = 7.1.

No relationship was found between the $pH_{mrs}$ and the morphometrical parameters. Spatially resolved bioluminescence and fluoroscopic imaging studies of pH values in tumour tissue showed a relationship with the distribution of perfused and non-perfused areas (Hossmann et al, 1992), but global $pH_{mrs}$ measurements failed in this study.

### ABBREVIATIONS

MRS, magnetic resonance spectroscopy; ivd, intervascular distance; $ivd_{200}$, intervascular distance larger than 200 μm; $Area_{100}$, non-perfused area fraction at a distance > 100 μm from a nearest perfused vessel; NTP, nucleoside triphosphate; $P_i$, inorganic phosphate; PME, phosphomonoesters; PDE, phosphodiesters; $pH_{mrs}$, pH measured with $^{31}$P-MRS (~ $pH_i$); $pH_i$, intracellular pH; $pH_e$, extracellular pH; $po_2$, oxygen tension; FID, free induction decay; PCr, phosphocreatine.

### ACKNOWLEDGEMENTS

The authors thank Dr L Hoofd and JH Creusen for helpful discussion, E van den Boogert, T Oostendorp, NEM Hagemeier,

J van Os and G Nachtegaal for technical assistance and J Koedam and colleagues of the Central Animal Laboratory for animal care. The NMR spectra were obtained using the Dutch HF–NMR facilities at the Biophysical Chemistry Department of Professor Dr CW Hilbers at the Nijmegen University. This study is supported by the Dutch Cancer Society.

### REFERENCES

Certaines de JD, Larsen VA, Podo F, Carpinelli G, Briot O and Henriksen O (1993) Review paper: in-vivo $_{31}$P MRS of experimental tumours. *NMR Biomed* **6**: 345–365

Dewhirst MW, Secomb TW, Ong ET and Gross JF (1994) Determination of local oxygen consumption rates in tumours. *Cancer Res* **54(13)**: 3333–3336

Eskey CJ, Korestky AP, Domach MM and Jain RK (1993) Role of oxygen vs. glucose in energy metabolism in a mammary carcinoma perfused ex vivo: direct measurement by $^{31}$P NMR. *Proc Natl Acad Sci* USA **90**: 2646–2650

Evelhoch JL, Sapareto SA, Nussbaum GH and Ackerman JJH (1986) Correlations between $^{31}$P NMR spectroscopy and $^{15}$O perfusion measurements in the RIF-1 murine tumor in-vivo. *Radiation Res* **106**: 122–131

Gerweck LE and Fellenz MP (1991) The simultaneous determination of intracellular pH and cell energy status. *Radiation Res* **125**: 257–261

Gerweck LE, Seneviratine T and Gerweck KK (1993) Energy status and radiobiological hypoxia at specified oxygen concentrations. *Radiation Res* **135**: 69–74

Groebe K and Vaupel P (1988) Evaluation of oxygen diffusion distances in human breast cancer xenografts using tumor-specific in-vivo data: role of various mechanisms in the development of tumor hypoxia. *Int J Radiat Oncol Biol Phys* **15**: 691–697

Hodgkiss RJ, Jones G, Long A, Parrick J, Smith KA, Stratford MRL and Wilson GD (1991) Flow cytometric evaluation of hypoxic cells in solid experimental tumours using fluorescence immunodetection. *Br J Cancer* **63**: 119–125

Hossmann KA, Linn F and Okada Y (1992) Bioluminescence and fluoroscopic imaging of tissue pH and metabolites in experimental brain tumours of Cat. *NMR Biomed* **5**: 259–264

Ikezaki K, Black KL, Conklin SG and Becker DP (1992) Histochemical evaluation of energy metabolism in rat glioma. *Neurol Res* **14(4)**: 289–293

Kallinowski F, Runkel S, Fortmeyer HP, Förster H and Vaupel P (1987) L-Glutamine: a major substrate for tumor cells in-vivo? *J Cancer Res Clin Oncol* **113**: 209–215

Kreuzer F (1982) Oxygen supply to tissues: the Krogh model and its assumptions. *Experientia* **38**: 1415–1426

Less JR, Skalak TC, Sevick EM and Jain RK (1991) Microvascular architecture in a mammary carcinoma: branching patterns and vessel dimensions. *Cancer Res* **51**: 265–273

Loesberg C, van Rooij H, Nooijen WJ, Meijer AJ and Smets LA (1990) Impaired mitochondrial respiration and stimulated glycolysis by m-iodobenzylguanidine (MIBG). *Int J Cancer* **46**: 276–281

Moon RB and Richards JH (1973) Determination of intracellular pH by 31P magnetic resonance. *J Biol Chem* **248**: 7276–7280

Okunieff PG, Koutcher JA, Gerweck L, McFarland E, Hitzig B, Urano M, Brady T, Neuringer L and Suit HD (1986) Tumor size dependent changes in a murine fibrosarcoma: use of in-vivo $^{31}$P NMR for non-invasive evaluation of tumor metabolic status. *Int J Radiat Oncol Biol Phys* **12**: 793–799

Okunieff P, Vaupel P Sedlacek R and Neuringer LJ (1989) Evaluation of tumor energy metabolism and microvascular blood flow after glucose or mannitol administration using $^{31}$P nuclear magnetic resonance spectroscopy and laser doppler flowmetry. *Int J Radiat Oncol Biol Phys* **16**: 1493–1500

Pianet I, Merle M, Labouesse J and Canioni P (1991) Phosphorus-31 nuclear magnetic resonance of C6 glioma cells and rat astrocytes: evidence for a modification of the longitudinal relaxation time of NTP and $P_i$ during glucose starvation. *Eur J Biochem* **195**: 87–95

Rhodes CG, Wise RJ, Gibbs JM, Frackowiak RS, Hatazawa J, Palmer AJ, Thomas DG and Jones T (1983) In-vivo disturbance of the oxidative metabolism of glucose in human cerebral gliomas. *Ann Neurol* **14(6)**: 614–626

Rijken PFJW, Bernsen HJJA and van der Kogel AJ (1995) Application of an image analysis system to the quantitation of tumor perfusion and vascularity in human glioma xenografts. *Microvascular Res* **50**: 141–153

Rofstad EK, Demuth P, Fenton BM and Sutherland RM (1988) $^{31}$P nuclear magnetic resonance spectroscopy studies of tumor energy metabolism and its relationship to intravessel oxyhemoglobin saturation status and tumor hypoxia. *Cancer Res* **48**: 5440–5446

Seo Y, Murahami M, Watari H, Imal LY, Yoshizaki K, Nishihawa H and Morimoto T (1983) Intracellular pH determination by $^{31}$P NMR technique. *J Biochem* **94**: 729–733

Stubbs M, Bhujwalla ZM, Tozer GM, Rodriques LM, Maxwell RJ, Morgan R, Howe FA and Griffiths JR (1992) An assessment of $^{31}$P MRS as a method of measuring pH in rat tumours. *NMR Biomed* **5**: 351–359

Tozer GM and Griffiths JR (1992) The contribution made by cell death and oxygenation to $^{31}$P MRS observations of tumour energy metabolism. *NMR Biomed* **5**: 279–289

Vaupel P (1974) Atemgaswechsel und Glucose-stoffwechsel von Implantations-tumoren (DS-Carcinosarkom) in-vivo. *Akademie der Wissenschaften und der Literatur, Mainz* **1**: 78–97

Vaupel P (1996) Microcirculation and blood flow: major determinants of tumor tissue oxygenation. In *rHErythropoietin in Cancer Supportive Treatment*, Smith JF, Boogaerts MA and Ehmer BRM (eds), pp. 206–213. Marcel Dekker: New York

Vaupel P, Okunieff P, Kallinowski F and Neuringer LJ (1989*a*) Correlations between $^{31}$P-NMR spectroscopy and tissue $O_2$ tension measurements in a murine fibrosarcoma. *Radiation Res* **120**: 477–493

Vaupel P, Kallinowski F and Okunieff P (1989*b*) Blood flow, oxygen and nutrient supply, and metabolic microenvironment of human tumors: a review. *Cancer Res* **49**: 6449–6465

Vaupel P, Kelleher DK and Engel T (1994) Stable bioenergetic status despite substantial changes in blood flow and tissue oxygenation in a rat tumour. *Br J Cancer* **69**: 46–49

Wendland MF, Sujata BI, Karen KF, Lam KN and James TL (1992) Correlations between in-vivo $^{31}$P MRS measurements, tumor size, cell survival, and hypoxic fraction in the murine EMT6 tumor. *Mag Res Med* **25**: 217–232

Yoshii Y and Sugiyama K (1988) Intercapillary distance in the proliferating area of human glioma. *Cancer Res* **48**: 2938–2941

© Cancer Research Campaign 1997